# A simplified approach to detect a significant carbon dioxide reduction by phytoplankton in lakes and rivers on a regional and global scale

**DOI:** 10.1007/s00114-020-01685-y

**Published:** 2020-06-23

**Authors:** Fabian Engel, Katrin Attermeyer, Gesa A. Weyhenmeyer

**Affiliations:** 1 Department of Ecology and Genetics/Limnology, Uppsala University, Norbyvägen 18D, 752 36 Uppsala, Sweden; 2 Present Address: WasserCluster Lunz – Biologische Station GmbH, Dr. Carl Kupelwieser Promenade 5, 3293 Lunz am See, Austria

**Keywords:** Phytoplankton, Global carbon cycle, Inland waters, Total organic carbon, CO_2_ dynamics, Chlorophyll *a*

## Abstract

Carbon dioxide (CO_2_) uptake by phytoplankton can significantly reduce the partial pressure of CO_2_ (*p*CO_2_) in lakes and rivers, and thereby CO_2_ emissions. Presently, it is not known in which inland waters on Earth a significant *p*CO_2_ reduction by phytoplankton is likely. Since detailed, comparable carbon budgets are currently not available for most inland waters, we modified a proxy to assess the *p*CO_2_ reduction by phytoplankton, originally developed for boreal lakes, for application on a global scale. Using data from 61 rivers and 125 lakes distributed over five continents, we show that a significant *p*CO_2_ reduction by phytoplankton is widespread across the temperate and sub-/tropical region, but absent in the cold regions on Earth. More specifically, we found that a significant *p*CO_2_ reduction by phytoplankton might occur in 24% of the lakes in the temperate region, and 39% of the lakes in the sub-/tropical region. We also showed that such a reduction might occur in 21% of the rivers in the temperate region, and 5% of the rivers in the sub-/tropical region. Our results indicate that CO_2_ uptake by phytoplankton is a relevant flux in regional and global carbon budgets. This highlights the need for more accurate approaches to quantify CO_2_ uptake by primary producers in inland waters, particularly in the temperate and sub-/tropical region.

**Electronic supplementary material:**

The online version of this article (10.1007/s00114-020-01685-y) contains supplementary material, which is available to authorized users.

## Introduction

To explain and predict matter fluxes between the Earth system components, land, ocean, and atmosphere, and their connection to the climate system, a comprehensive understanding of biogeochemical processes in lakes and running waters is required (Ward et al. 2017). In recent years, attempts to map the characteristics of many of the 117 million lakes on Earth (e.g., Chen et al. 2015) and to quantify riverine carbon export from land to sea on a continental and global scale (e.g., Li et al. 2017) revealed regional differences in inland water carbon dynamics. Since carbon cycling in inland waters is spatially highly variable, a geographic perspective is necessary to understand the role of lakes and running waters as part of the Earth system (Seekell et al. 2018).

Carbon cycling in inland waters refers to the transformation of carbon between its different forms, often the transformation of inorganic carbon into organic carbon and vice versa. On a global scale, it has been observed that the gaseous share of inorganic carbon in lakes, measured as partial pressure of CO_2_ (*p*CO_2_), generally increases towards regions with higher temperatures (Marotta et al. 2009). However, the increasing *p*CO_2_ towards warmer regions contradicts the greater CO_2_ uptake by phytoplankton in warmer regions (Lewis Jr 2011). Hence, the *p*CO_2_ and the extent of CO_2_ uptake by phytoplankton in lake water seem to be decoupled over large spatial scales. Yet, a direct comparison between the CO_2_ uptake by phytoplankton in inland waters and the *p*CO_2_ in the water column is not available on a global scale. Furthermore, the *p*CO_2_ in global studies has generally not been measured in the field, but been calculated based on water temperature, pH, and alkalinity measurements or estimated from dissolved organic carbon (DOC) concentration. Thus, substantial errors, particularly in acidified waters, have been reported (e.g., Golub et al. 2017). Consequently, *p*CO_2_ patterns on a global scale are still relatively inaccurate, which might explain the apparent geographic decoupling of patterns in the *p*CO_2_ and CO_2_ uptake by phytoplankton.

**Fig. 1:**
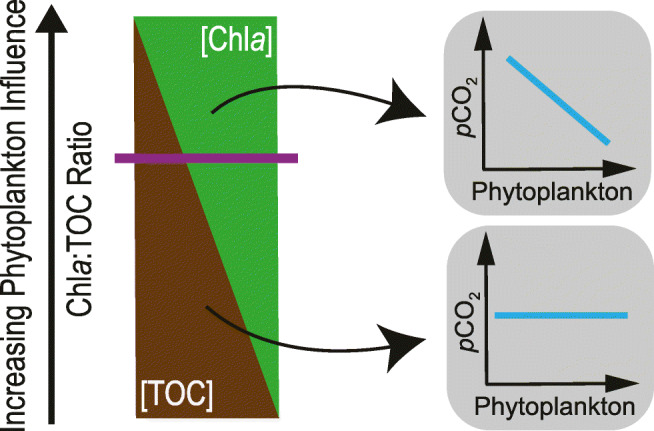
Conceptual figure illustrating the use of the chlorophyll *a* to total organic carbon concentration ratio (Chl*a*:TOC ratio) as a proxy for the phytoplankton influence on the partial pressure of CO_2_ (*p*CO_2_) in the water column. The proxy had originally been developed for boreal lakes (Engel et al. 2019) and is here modified for application on a global scale. The higher the ratio, the more likely is a significant *p*CO_2_ reduction by phytoplankton (right panel). In water bodies with a ratio exceeding a certain threshold value (indicated by the horizontal line in the left panel), a significant *p*CO_2_ reduction by phytoplankton can be expected

A better understanding of the influence of phytoplankton on the *p*CO_2_ in lakes and rivers over large spatial scales would require a large number of inland water carbon budgets. These data are presently not available. To overcome this limitation, we tested here a simple proxy to assess the phytoplankton influence on the *p*CO_2_ in inland waters on a global scale (Fig. 1). We also outline future research directions to progress knowledge on CO_2_ uptake by primary production in lakes and rivers over large spatial scales.

## Current knowledge

Inland water carbon budgets on local, regional, and global scales have been extensively described. In this section, we do not aim to provide a comprehensive literature review on carbon budgets but instead focus on challenges in assessing the influence of CO_2_ uptake by primary production on inland water carbon budgets over large geographic scales.

In whole-lake carbon budgets of single lakes, primary production in the pelagic (phytoplankton), benthic (algae), and littoral habitat (macrophytes and periphyton) might be quantified separately (e.g., Andersson and Kumblad 2006). Alternatively, it can be estimated using the diel oxygen (or CO_2_) method that integrates CO_2_ uptake by all primary producers (e.g., Chmiel et al. 2016). When using mass balance approaches, only carbon inflows and outflows of a lake are described, but lake internal carbon transformation is not quantified (Andersson and Sobek 2006). Consequently, CO_2_ uptake by primary production commonly remains undescribed in mass balance approaches (e.g., Pacheco et al. 2014).

The biggest constraints for comparing whole-lake carbon budgets are the use of different methodologies to compile such budgets (e.g., methods for measuring carbon fluxes, temporal extent of the studies), and differences in reporting results (e.g., type of fluxes reported, units used). These discrepancies make it difficult to compare the influence of CO_2_ uptake by primary production on carbon budgets between lakes. To our knowledge, no standardized guideline on how to compile a lake carbon budget exists. Hence, the comparison of different studies and estimation of the influence of primary producers over large spatial scales is still challenging.

To estimate carbon budgets for inland waters on a regional and global scale, measurements from a fraction of inland waters within a region might be scaled up (e.g., Molot and Dillon 1996). However, studies on a regional and global scale mostly rely on modeling approaches (e.g., Algesten et al. 2004; Lewis Jr 2011). Assessing the phytoplankton influence on inland water carbon budgets on a regional and global scale is yet limited, because in situ measurements of biological properties, like phytoplankton biomass or primary production, are time-consuming and costly. Some advancements have recently been made. Chlorophyll *a* concentration (Chl*a*; a proxy for phytoplankton biomass) can, for example, be sensed remotely (Sayers et al. 2015) and used to quantify primary production (Cole and Cloern 1987). Remote sensing of Chl*a* in inland waters is, however, accompanied by high measurement uncertainties and strong limitations related to challenges in detecting small water bodies (Sayers et al. 2015). Consequently, estimating the importance of CO_2_ uptake by phytoplankton in regional and global budgets has still to rely on in situ measurements of Chl*a* and on modeling approaches.

In a recent analysis that used Chl*a* measurements from 1157 water bodies in the USA and *p*CO_2_ calculated from pH, alkalinity, and water temperature, Chl*a* was found to have a significant effect on *p*CO_2_ in 68% of the studied inland waters (Lapierre et al. 2017). This indicates that CO_2_ uptake by phytoplankton might significantly influence the CO_2_ balance of lakes on a regional scale. However, in carbon budgets that integrate aquatic and terrestrial components on a regional scale, CO_2_ uptake by primary production in inland waters has often been neglected (e.g., Öquist et al. 2014). This is probably due to a focus on carbon inflow, outflow, and storage on land.

Recently, the organic carbon budget of global reservoirs has been modeled, including CO_2_ uptake by primary production (Maavara et al. 2017). In that study, primary production was assumed to be phosphorus limited and was estimated as a function of dissolved phosphorus concentration as well as Chl*a* and the chlorophyll-specific carbon fixation rate corrected for water temperature (Maavara et al. 2017). Furthermore, a recent literature review on cross-ecosystem carbon flows on a global scale summarized the existing data on CO_2_ uptake by primary production in lakes and streams (Gounand et al. 2018). Nevertheless, a large number of comparable inland water carbon budgets are needed to assess the role of CO_2_ uptake by primary production, which are currently not available. Thus, we demonstrate here that easily available water physico-chemical and biological variables can provide a first estimate of spatial variation in the potential for a significant *p*CO_2_ reduction by phytoplankton in lakes and rivers.

## A simple proxy to identify a *p*CO_2_ reduction by phytoplankton on a global scale

The phytoplankton carbon share in total organic carbon (TOC; phytoplankton share in TOC abbreviated as C_phyto_:TOC ratio) has recently been shown to be a useful proxy to detect boreal lakes in which the *p*CO_2_ might be significantly reduced by phytoplankton (Engel et al. 2019). The C_phyto_:TOC ratio was used to divide lakes into those with and without a significant *p*CO_2_ reduction by phytoplankton (Fig. 1). In lakes with a high C_phyto_:TOC ratio, a significant (*P* < 0.05) negative relationship between phytoplankton biomass and the *p*CO_2_ in the water column was observed. These lakes were considered to have the potential for a significant *p*CO_2_ reduction by phytoplankton. For lakes that showed a low C_phyto_:TOC ratio, no relationship between phytoplankton biomass and the *p*CO_2_ was detected, indicating that *p*CO_2_ reduction by phytoplankton was not the dominant process controlling the *p*CO_2_ in these lakes (for more details, see Supplementary material).

Since phytoplankton carbon data is not available for most inland waters, we modified the proxy (C_phyto_:TOC ratio) and used Chl*a* instead of phytoplankton carbon; i.e., we calculated the mass ratio of Chl*a* to TOC (Chl*a*:TOC ratio). Chl*a* has before been used in a similar proxy, the ratio of Chl*a* to light extinction (light extinction as proxy for humic substances) (Kosten et al. 2010). In lakes with a low ratio, the *p*CO_2_ tended to be high. Therefore, we tested if the C_phyto_:TOC ratio can be replaced by the Chl*a*:TOC ratio, and validated its use on a global scale. A Chl*a*:TOC ratio of 2.0 × 10^−3^ was identified as the threshold value for identifying water bodies with a significant *p*CO_2_ reduction by phytoplankton (Figs. S1 and S2; for more details, see Supplementary material). We acknowledge that the use of the Chl*a*:TOC ratio can only provide a rough estimate of spatial variation in the potential for a significant *p*CO_2_ reduction by phytoplankton which is further discussed below.

### Application of the proxy on a global scale

We used data on Chl*a* (in μg l^−1^) and TOC (in mg l^−1^) from 125 lakes and reservoirs (hereafter lakes) as well as 61 rivers (with mean annual discharge > 2 m^3^ s^−1^) distributed around the globe (Tabs. S1 and S2). The literature was selected through a Google Scholar and Web of Science search using the search terms “chlorophyll a” and “total organic carbon” plus the terms “lake” or “river.” Besides, literature was also searched through citations in relevant studies. A more directed search was performed for data from large river systems as well as for data from tropical systems, since data from this region were less frequently retrieved in the Google Scholar and Web of Science search.

We classified the lakes and rivers according to their geographic position within three a priori defined geographic regions termed “cold,” “temperate,” and “sub-/tropical.” This division allowed us to explore differences in the Chl*a*:TOC ratio between these regions. The three geographic regions were derived by combining the 14 biomes described in Olson et al. (2001) into three regions. The biomes “Tundra” and “Boreal Forests/Taiga” were combined to the cold region. The biomes “Temperate Broadleaf and Mixed Forests,” “Temperate Coniferous Forests,” “Temperate Grasslands, Savannahs, and Scrubland,” “Montane Grasslands and Scrublands,” and “Mediterranean Forests, Woodlands, and Scrublands” were combined to the temperate region. The remaining biomes were included in the sub-/tropical region.

Our dataset contained 11 river sites in the cold region, 29 in the temperate region, and 21 in the sub-/tropical region as well as 14 lake sites in the cold region, 88 in the temperate region, and 23 in the sub-/tropical region. For studies reporting similar Chl*a*/TOC for more than 25 lakes from one province, or for studies reporting only an average value for a large number of lakes, we used a representative composite value (i.e., mean value) (Tab. S2). These were studies describing 204, 78, 70, 46, and 34 lakes in the cold region, and one study with 31 lakes in the sub-/tropical region. Except for published literature, data for six Swedish rivers were taken from the Swedish national inland water inventory program and data for one German river from the monitoring program of the German federal state of Rhineland-Palatinate (Tab. S1). These river data can be freely accessed at http://miljodata.slu.se/mvm/ and at http://www.geoportal-wasser.rlp.de/servlet/is/2025/.

For studies in which only DOC concentrations were given (8 lake sites in the cold region, 31 in the temperate region, 20 in the sub-/tropical region, and one river site in the cold region), we calculated TOC by dividing DOC by 0.9. The ratio of DOC to POC in unproductive and moderately productive lakes commonly lies around 10:1 but can fluctuate, for example, during algal blooms (Wetzel 2001). A conversion factor of 10:1 has earlier been used in studies on a global scale (Chen et al. 2015; Sobek et al. 2007), which is further discussed below.

We tested the data for normality using a Shapiro-Wilk test. Since the data did not follow a normal distribution, we used non-parametric tests. We used the Kruskal-Wallis test followed by pairwise comparison of groups using a Wilcoxon rank-sum test with Holm-Bonferroni correction (Holm 1979) to test for differences in the Chl*a*:TOC ratio between regions for lakes and rivers. We further used the Wilcoxon test to compare the Chl*a*:TOC ratio between lakes and rivers per region. All tests were performed with the software program JMP, version 13.0.1 (SAS Institute Inc., Cary, NC, USA).

### Spatial variation in the Chl*a*:TOC ratio in lakes

**Fig. 2:**
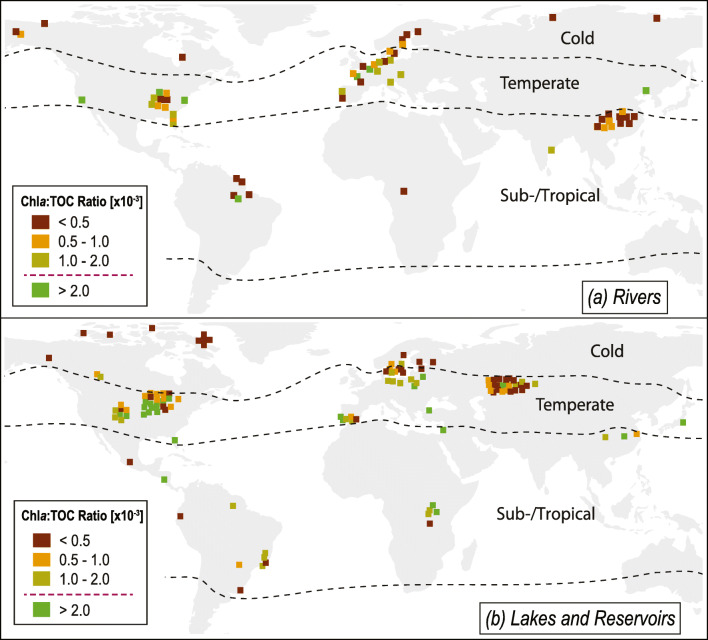
Global distribution of the Chl*a*:TOC ratio (mass ratio of the chlorophyll *a* to total organic carbon concentration) in rivers and lakes/reservoirs. The dashed lines indicate the boundaries between the cold and the temperate region as well as between the temperate and the sub-/tropical region. **a** Chl*a*:TOC ratio for 61 river sites. **b** Chl*a*:TOC ratio for 125 lake and reservoir sites. A greater ratio indicates a higher potential for a significant *p*CO_2_ (partial pressure of CO_2_) reduction by phytoplankton. Above a Chl*a*:TOC ratio of 2.0 × 10^−3^ (threshold marked in the legend with dashed line), a significant *p*CO_2_ reduction by phytoplankton can be expected. For a more detailed description of the threshold, see Supplementary material and Fig. S1. Data for chlorophyll *a* concentration and total organic carbon concentration were collected from the literature listed in Tables S1 and S2. Graphical overlaps in map areas with many data points were reduced by dispersing the symbols

**Fig. 3:**
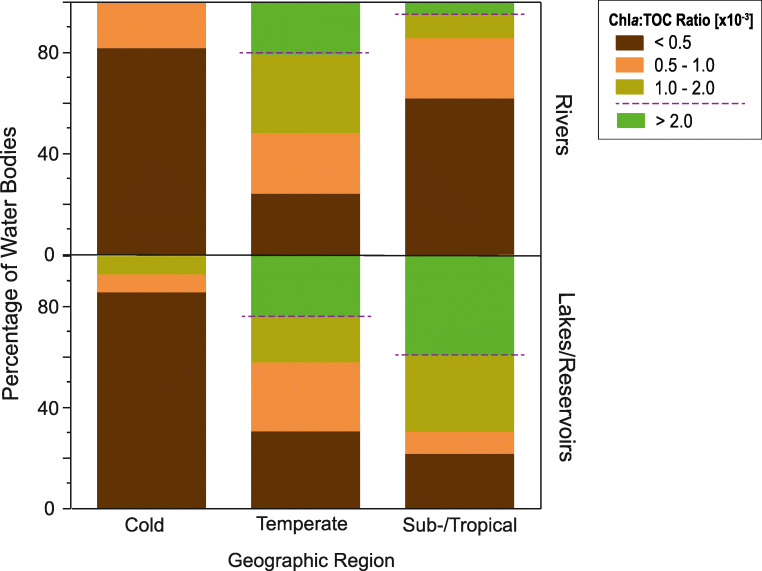
Percentage of rivers and lakes/reservoirs with a Chl*a*:TOC ratio (mass ratio of the chlorophyll *a* to total organic carbon concentration) of < 0.5 × 10^−3^, 0.5 × 10^−3^ – 1 × 10^−3^, 1 × 10^−3^ – 2 × 10^−3^, and > 2 × 10^−3^ per geographic region. A greater ratio indicates a higher potential for a significant *p*CO_2_ (partial pressure of CO_2_) reduction by phytoplankton. Above a Chl*a*:TOC ratio of 2.0 × 10^−3^ (threshold marked with dashed line), a significant phytoplankton influence on the *p*CO_2_ can be expected. For a more detailed description of the threshold, see Supplementary material and Fig. S1. Data for chlorophyll *a* concentration and total organic carbon concentration were collected from the literature listed in Tabs. S1 and S2

The Chl*a*:TOC ratio in lakes varied significantly between regions (Kruskal-Wallis test, *P* < 0.0001; Figs. 2 and 3). Chl*a*:TOC ratios in both the temperate and sub-/tropical region were significantly higher than in the cold region (pairwise Wilcoxon test, *P* < 0.001, Holm-Bonferroni corrected for three observations; Figs. 2 and 3). This indicates that the Chl*a*:TOC ratio in lakes increases from the cold to the temperate region. This pattern is expected since it corresponds to an increasing phytoplankton primary production following an overall latitudinal gradient in temperature, light, and nutrient availability (Lewis Jr 2011). Accordingly, spatial variation in the phytoplankton to TOC ratio in boreal lakes has earlier been found to be best predicted by nutrient availability, i.e., total phosphorus and total nitrogen concentration (Engel et al. 2019).

We found no significant difference in the Chl*a*:TOC ratio between the temperate and sub-/tropical region (pairwise Wilcoxon test, *P* > 0.05, Holm-Bonferroni corrected for three observations; Figs. 2 and 3). Most TOC data for lakes in the sub-/tropical region in our study were estimated from DOC measurements, neglecting a potentially increasing share of particulate organic matter in TOC in the sub-/tropical region. This might have led to an underestimation of TOC, and thus an overestimation of the Chl*a*:TOC ratio in 20 out of 23 lakes located in the sub-/tropical region. Thus, *p*CO_2_ patterns in lakes of this region need further investigation in order to draw final conclusions.

### Spatial variation in the Chl*a*:TOC ratio in rivers

The Chl*a*:TOC ratio in rivers varied significantly between regions (Kruskal-Wallis test, *P* < 0.001; Figs. 2 and 3). The ratio was significantly higher in the temperate region than in both the cold and sub-/tropical region (pairwise Wilcoxon test, *P* < 0.05, Holm-Bonferroni corrected for three observations; Figs. 2 and 3). This might be a consequence of high population densities over large parts of the temperate region (Center for International Earth Science Information Network - CIESIN - Columbia University 2016). River systems in densely populated areas are highly impacted by damming and eutrophication, which both can increase riverine phytoplankton production (Wetzel 2001).

We found no significant difference in the Chl*a*:TOC ratio between the cold and sub-/tropical region (pairwise Wilcoxon test, *P* > 0.05, Holm-Bonferroni corrected for three observations; Figs. 2 and 3). The lower average Chl*a*:TOC ratios in the cold and sub-/tropical compared with the temperate region might be a consequence of high terrestrial organic matter loads in rivers of the cold and sub-/tropical region (Li et al. 2017). High terrestrial organic matter loads can lead to increased light limitation of phytoplankton, and thus cause low Chl*a*:TOC ratios. Increased light limitation might also lead to increased Chl*a* per cell in order to adapt to light limitation. In this case, we would slightly overestimate the phytoplankton share in TOC using the Chl*a*:TOC ratio as a proxy.

### Differences in the Chl*a*:TOC ratio between lakes and rivers across regions

We found divergent patterns in the Chl*a*:TOC ratio in lakes and rivers across regions (Fig. 3). For rivers, the highest median Chl*a*:TOC ratio was found in the temperate region (1.0 × 10^−3^ ± 0.7 × 10^−3^, median ± median absolute deviation), while the highest median ratio for lakes was in the sub-/tropical region (1.7 × 10^−3^ ± 1.2 × 10^−3^). The increasing Chl*a*:TOC ratio towards warmer regions is expected since primary production in such regions is often high (Lewis Jr 2011). High phytoplankton production in lakes has earlier been shown to have the potential to reverse lake carbon budgets, turning them into carbon sinks (Pacheco et al. 2014).

The result that phytoplankton might significantly reduce the *p*CO_2_ in around 20% of the temperate river systems (Fig. 3) has until now not been considered in the exploration of inland water carbon cycling on a global scale. Our results are in line with a recent study that showed a strong negative relation between Chl*a* and *p*CO_2_ in a basin-scale survey along a Korean river (Yoon et al. 2017). However, CO_2_ uptake by phytoplankton in rivers is likely a minor flux compared with the global CO_2_ emissions from running waters on Earth (Gounand et al. 2018; Lauerwald et al. 2015). Nevertheless, our results suggest that it might still be relevant for the CO_2_ dynamics in rivers with high Chl*a*:TOC ratios.

While the Chl*a*:TOC ratio in the sub-/tropical region was significantly higher in lakes than in rivers (Wilcoxon test, *P* < 0.01; Fig. 3), we did not find any significant differences in the ratio between lakes and rivers in both the temperate and cold region (Wilcoxon test, *P* > 0.05; Fig. 3). Differences in the Chl*a*:TOC ratio between lakes and rivers within the same region can be attributed to different morphological and ecological characteristics of lentic and lotic ecosystems. Differences in the ratio might also be a consequence of human influences on inland waters that might affect lakes and rivers within the same geographic region differently. The combined effect of eutrophication and damming might, for example, result in a comparatively stronger effect on primary production in rivers than just eutrophication in lakes.

### Conceptualization of global patterns in *p*CO_2_ reduction by phytoplankton

**Fig. 4:**
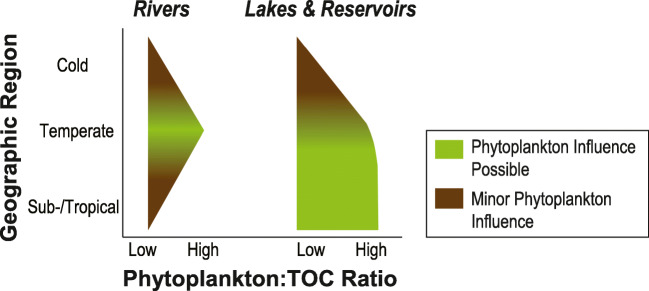
Conceptual figure showing the effect of the location of rivers and lakes/reservoirs on the size of the phytoplankton:TOC ratio (in our study evaluated as mass ratio of chlorophyll *a* to total organic carbon concentration). In regions where high phytoplankton:TOC ratios (green shaded) are common, the *p*CO_2_ in inland waters might be significantly reduced by CO_2_ uptake by phytoplankton. In regions with low phytoplankton:TOC ratios (brown shaded), the influence of phytoplankton is minor, and CO_2_ dynamics might be dominated by mineralization of organic matter or hydrologic inorganic carbon inflows from terrestrial ecosystems. The conceptual figure is based on the results from this study and the data shown in Figs. 2 and 3, and Tabs. S1 and S2

In lakes and rivers with a Chl*a*:TOC ratio > 2.0 × 10^−3^, a significant *p*CO_2_ reduction by phytoplankton can be expected (Figs. S1 and S2; Supplementary material). In the cold region, we did not find any Chl*a*:TOC ratio > 2.0 × 10^−3^, and in 84% of the water bodies, we observed a Chl*a*:TOC ratio < 0.5 × 10^−3^ (Fig. 3). These results suggest that CO_2_ uptake by phytoplankton was low in relation to CO_2_ production by internal mineralization of organic matter and hydrologic CO_2_ inflow. Consequently, phytoplankton had a minor influence on the *p*CO_2_ in both lakes and rivers in the cold region (Fig. 4). This is consistent with metabolic theory that predicts a greater temperature dependence of photosynthesis compared with respiration (Yvon-Durocher et al. 2010). Thus, differences in the Chl*a*:TOC ratio between geographic regions are to be expected. Cold temperatures, together with unfavorable light conditions at higher latitudes, can inhibit phytoplankton growth and result in a lower phytoplankton influence.

In the temperate region, 23% of lakes and 20% of rivers in our dataset showed Chl*a*:TOC ratios > 2.0 × 10^−3^ (Fig. 3). This suggests that CO_2_ uptake by phytoplankton might significantly reduce the *p*CO_2_ in around one-fifth of lakes and rivers within this region. Chl*a* has recently been shown to be a useful predictor for modeling lake water *p*CO_2_ in some parts of the temperate region (Lapierre et al. 2017). Furthermore, lakes with high primary production have been found to be associated with lower *p*CO_2_ (Balmer and Downing 2011).

In the sub-/tropical region, differences between lakes and rivers were large, with 5% of the rivers and 39% of the lakes exceeding a Chl*a*:TOC ratio of 2.0 × 10^−3^ (Fig. 3). The proportion of phytoplankton carbon that is mineralized by pelagic bacteria has earlier been found to be lower in tropical compared with non-tropical inland waters (Roland et al. 2010). This finding, together with high Chl*a*:TOC ratios in many sub-/tropical lakes, suggests that phytoplankton might significantly reduce the lake water *p*CO_2_, and CO_2_ uptake by phytoplankton is likely an important carbon flux in many lakes of this region.

We show here spatial variation in the potential for a significant *p*CO_2_ reduction by phytoplankton in 125 lakes and 61 rivers in the cold, temperate, and sub-/tropical region by applying the Chl*a*:TOC ratio on a global scale (Figs. 2 and 3). In total, 24% of lakes and 11% of rivers in our dataset showed conditions that make a significant *p*CO_2_ reduction by phytoplankton likely (i.e., a Chl*a*:TOC ratio > 2.0 × 10^−3^). The global average Chl*a*:TOC ratio was nearly twice as high in lakes (1.1 × 10^−3^ ± 0.6 × 10^−3^) as in rivers (0.6 × 10^−3^ ± 0.3 × 10^−3^), but inter-regional differences were even larger (the median difference between the cold and the temperate region for lakes and rivers combined was 0.9 × 10^−3^ ± 0.7 × 10^−3^). At the same time, the Chl*a*:TOC ratio was highly variable within any one region; thus, the intra-regional variability of *p*CO_2_ reduction by phytoplankton is likely to be high.

## Future research directions

Using the Chl*a*:TOC ratio as a simple proxy, we identified regions on Earth in which CO_2_ uptake by phytoplankton might significantly reduce the *p*CO_2_ in lakes and rivers. To our knowledge, this is the first global inventory of this kind. To determine the applicability of the Chl*a*:TOC ratio more broadly, an even larger data set across time and space is needed. Below, we suggest future research directions to address the role of primary production in inland water carbon budgets on a global scale.

Firstly, the use of Chl*a* as a proxy for phytoplankton biomass or phytoplankton carbon content comes with uncertainties. Related to the physiology of algae, Chl*a* can vary independently of biomass. The ratio of Chl*a* to phytoplankton carbon is, for example, known to vary in different phytoplankton taxa between 1.22 and 6.08% (Riemann et al. 1989) or 0.88 and 3.85% (Yacobi and Zohary 2010). Moreover, Chl*a* in inland waters is frequently quantified measuring the fluorescence yield of Chl*a*, which varies at constant Chl*a* depending on environmental conditions (nutrients, light), and is accompanied by measurement errors depending on the instrumentation and procedure used (Proctor and Roesler 2010).

Secondly, characterizing an ecosystem carbon flux (CO_2_ uptake by phytoplankton) using an ecosystem state variable (like Chl*a*) needs consideration. Phytoplankton productivity can vary widely at constant biomass, depending on the light intensity reaching the water surface, light absorption by the water (depending on turbidity), variation in Chl*a*, and the efficiency with which photosynthetic products are produced in the cell (Wetzel 2001). Since carbon flux measurements to analyze spatial variation over large geographic regions are currently lacking, it can, as suggested by Soranno et al. (2019), for the moment be assumed that spatial variation in ecosystem states (e.g., Chl*a* or TOC) will apply similarly to carbon fluxes. As flux measurements become more widely available, for example, because of technological development or international cooperation, those should be used.

Thirdly, a substantial share of primary production in rivers is related to benthic algae or macrophytes (Vis et al. 2007). Phytoplankton production in rivers can be replaced by macrophytes (Ibáñez et al. 2012), resulting in an underestimation of the potential for *p*CO_2_ reduction by primary production when using the Chl*a*:TOC ratio as a proxy. Hence, future studies should consider all primary producers in the studied ecosystem. A dominance of benthic primary production or primary production by macrophytes might have a lower influence on CO_2_ dynamics in lakes because of their often greater water depth and volume compared with rivers. In shallow lakes, however, benthic primary production can be an important carbon flux (e.g., Vadeboncoeur et al. 2008). Benthic primary production is not considered in our study since we focused on phytoplankton and used Chl*a* measurements from the pelagic zone. Likewise, in shallow temperate lakes and small humic lakes, CO_2_ uptake by macrophytes has been shown to play an important role in the carbon budget (e.g., Vesterinen et al. 2016). To assess the role of CO_2_ uptake by primary production more accurately on a global scale, the growing amount of metabolism data might be used (e.g., Appling et al. 2018). Quantifying primary production using metabolism calculations (Odum 1956; Vachon et al. 2020) has the advantage of integrating all forms of primary production, not only considering CO_2_ uptake by phytoplankton in the pelagic zone. However, when using aquatic metabolism data, a higher measuring frequency than is typically used for phytoplankton biomass may be necessary, because metabolism is more variable on short time scales (Wetzel 2001). Furthermore, metabolism data does not directly indicate if the *p*CO_2_ is significantly reduced by primary production.

Fourthly, our approach does not consider temporal variation and is limited by data availability in different regions on Earth. We used Chl*a* and TOC from available published literature, which implies that different continents and regions are not equally represented. This is a common bias when studying inland waters on a global scale (Stanley et al. 2019). Usually, there is also a trade-off between temporal and spatial resolution, which explains why studies on a global scale often have a limited temporal resolution. Recently, it has been suggested that spatial variation in lake Chl*a* exceeds temporal variation within a macroscale (1000 km) considering summer measurements (Soranno et al. 2019). This suggests that the spatial patterns presented in our study might be robust towards variation in sampling time. However, differences in CO_2_ uptake by primary production between summer and winter are large in the temperate and cold region and should be quantified on large spatial scales. Since studies on a large spatial scale often have to rely on surface measurements, we lack vertical profiles for Chl*a* and TOC. Future studies should include some water column integrated Chl*a* and TOC and assess the role of vertical differences in the Chl*a*:TOC ratio on the predicted phytoplankton influence on the *p*CO_2_. Hence, future studies should improve the spatial as well as temporal resolution of the data used.

Fifthly, future research should assess human disturbances that might alter CO_2_ uptake by primary production in inland waters. The degree and direction of change caused by such disturbances are currently highly uncertain, and changes will likely affect inland waters in different regions differently (Kraemer et al. 2017). Browning of lakes across the northern hemisphere has, for example, been suggested to decrease primary production and phytoplankton biomass due to light limitation (Hessen et al. 2017). In nutrient-poor lakes, however, browning can also stimulate whole-lake primary production due to increased nutrient availability (Seekell et al. 2015). Changes in land cover such as an increase in agricultural land can boost primary production and cause major water quality problems in inland waters due to increased nutrient inputs from agricultural runoff (Knoll et al. 2003). An increase in atmospheric CO_2_ has been suggested to increase phytoplankton productivity in some lakes (e.g., Kragh and Sand-Jensen 2018), even if phytoplankton growth in inland waters is usually not considered CO_2_ limited. Unexpectedly, warming does not seem to be associated with a general increase in primary production in lakes (e.g., Schindler et al. 1990), and streams (e.g., Demars et al. 2016), but changes in *p*CO_2_ reduction by phytoplankton under a warming climate have to be further assessed.

Sixthly, the fate of carbon fixed by primary production in lakes and rivers has not been quantified on large spatial scales. Autochthonously produced organic carbon might either be re-mineralized to inorganic carbon or buried in sediments and thereby sequestered. Recent studies have shown that only about one-third of the sedimented carbon in arctic and boreal lakes is of autochthonous origin (Guillemette et al. 2017). Eutrophic lakes with high primary production have been suggested to act as carbon sinks, where large amounts of autochthonously fixed carbon are buried in sediments (Flanagan et al. 2006). It is likely that the fate of carbon fixed by primary production varies widely between lakes and rivers and between different regions. Thus, to integrate CO_2_ uptake by primary production fully into carbon budgets on large spatial scales, the fate of autochthonously fixed carbon needs to be unraveled.

Overall, we suggest quantifying CO_2_ uptake by primary producers in regional and global inland water carbon budgets more accurately. The simple proxy presented here can be a first step towards this. A further step might be classifying lakes and rivers according to their position along a continuum of functional lake/river types in carbon cycling (e.g., Engel et al. 2018; Savoy et al. 2019), and subsequently assigning a simplified carbon budget to each lake/river, according to its size, location, and functional type. To assign functional types to lakes/rivers, easy proxies like the Chl*a*:TOC ratio might be used, but further proxies that show the extent of other important carbon fluxes should be used to make the classification more accurate. To assign simplified carbon budgets to each lake/river, default carbon budgets for each position along the continuum of functional lake/river types should be defined. Those should be based on detailed, comparable, field-based carbon budgets from lakes and rivers from all regions on Earth.

## Electronic supplementary material

ESM 1 (PDF 748 kb)

## Data Availability

The data was retrieved from published literature or publicly available databases. Data and sources are provided in the supplementary material. Correspondence and requests for materials should be addressed to Fabian.Engel@ebc.uu.se.
