# Supplementary material for: A simplified approach to detect a significant carbon dioxide reduction by phytoplankton in lakes and rivers on a regional and global scale
Source: Naturwissenschaften. 2020 Jun 23;107(4):29. doi: 10.1007/s00114-020-01685-y (PMC7311510; doi:10.1007/s00114-020-01685-y)
Supplement: Supplementary file 1 — (PDF 748 kb) [file 114_2020_1685_MOESM1_ESM.pdf]

Supplementary Material

A simplified approach to detect a significant carbon dioxide reduction by phytoplankton in lakes and rivers on a regional and global scale

Fabian Engel[1*], Katrin Attermeyer[1,2], Gesa A. Weyhenmeyer[1]

[1]*Department of Ecology and Genetics/Limnology, Uppsala University, Norbyvägen 18D, 752 36 Uppsala, Sweden.*

[2]*current address: WasserCluster Lunz – Biologische Station GmbH, Dr. Carl Kupelwieser Promenade 5, 3293 Lunz am See, Austria*

**e-mail: Fabian.Engel@ebc.uu.se, phone: +46-18-471 2706*

Calibration and validation of the Chl*a*:TOC ratio as a proxy for a significant pCO_2 reduction by phytoplankton

Data and Analysis

To identify lakes and rivers with a significant reduction in the partial pressure of CO_2 (pCO_2) by phytoplankton, we used the approach of the phytoplankton carbon share in total organic carbon (C_{phyto}:TOC ratio), which has originally been developed for boreal lakes (Engel et al. 2019). Since sufficient data on phytoplankton carbon were not available on a global scale, we first evaluated whether the C_{phyto}:TOC ratio can be replaced by the Chl*a*:TOC ratio. We related C_{phyto}:TOC to Chl*a*:TOC, using the dataset of 126 Swedish boreal lake sites from Engel et al. (2019). The data can be freely accessed at http://miljodata.slu.se/mvm/. We applied a Kendall`s tau correlation due to the non-normal distribution of the data, tested with a Shapiro-Wilk test.

Subsequently, we validated the suitability of the Chl*a*:TOC ratio that had only been tested for boreal lakes, for inland waters in the temperate and sub-/tropical region. For the validation, we collected data on pCO_2, TOC, and Chl*a* from 56 temperate, and 16 sub-/tropical lakes (Tab. S3), all showing high Chl*a*:TOC ratios. Due to the non-normal distribution of the data we log-transformed it, and then related Chl*a* to pCO_2 by performing a linear regression. All tests were performed using JMP, version 13.0.1 (SAS Institute Inc., Cary, NC, U.S.A.).

Calibration and validation results

Relating the C_{phyto}:TOC ratio to the Chl*a*:TOC ratio, using data from 126 Swedish lake sites, we found a highly significant relationship (Kendall`s tau = 0.71, $P < 0.0001$, n = 126; Fig. S1). This strong relation suggests that the C_{phyto}:TOC ratio can be replaced by the Chl*a*:TOC ratio.

A C_{phyto}:TOC ratio of > 5%, which has been identified as threshold value for a significant pCO_2 reduction by phytoplankton (Engel et al. 2019), corresponded to a Chl*a*:TOC ratio of > 2.0×10^{-3} (Fig. S1).

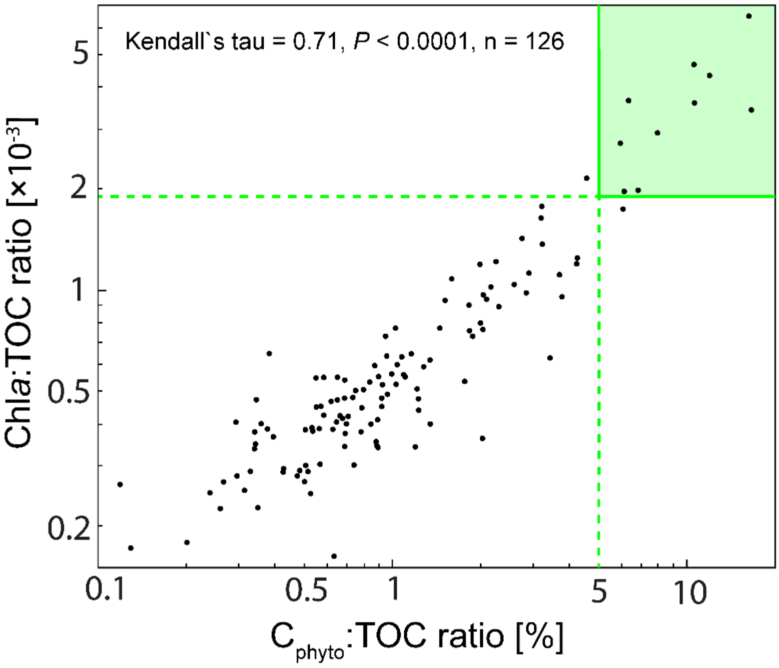

Figure S1. Relationship between the C_{phyto}:TOC ratio (phytoplankton carbon share in total organic carbon) and the Chla:TOC ratio (mass ratio of the chlorophyll a concentration to total organic carbon) at 126 Swedish lake sites. Shown are long-term median August values per lake site from surface water samples collected between 1992 and 2012. Both axes are displayed on a logarithmic scale. The green (dashed) lines indicate the C_{phyto}:TOC ratio threshold above which phytoplankton significantly reduced the lake water pCO_2 according to Engel et al. (2019). The C_{phyto}:TOC ratio threshold of 5 % corresponded to a Chla:TOC ratio threshold of around 2×10^{-3}. The green shaded area indicates lakes in which the pCO_2 might be significantly reduced by phytoplankton.

Subsequently, we tested whether the Chl*a*:TOC ratio is also applicable as a proxy for the phytoplankton influence on lake water pCO_2 in the temperate and sub-/tropical region. Selecting lakes with a Chl*a*:TOC ratio > 2.0×10^{-3} located in the temperate and sub-/tropical region, and relating Chl*a* to pCO_2 in these lakes, we were able to confirm a significant negative relationship between Chl*a* and pCO_2 (R^2 = 0.27, P < 0.0001, n = 72; Fig. S2). These results indicate that the pCO_2 is reduced by CO_2 uptake by phytoplankton in lakes showing a Chl*a*:TOC ratio > 2.0×10^{-3}. Thus, we suggest that the Chl*a*:TOC ratio is a useful simple proxy to identify inland

waters in which the pCO_2 might be significantly reduced by phytoplankton not only in the boreal zone, but also in the temperate, and sub-/tropical region.

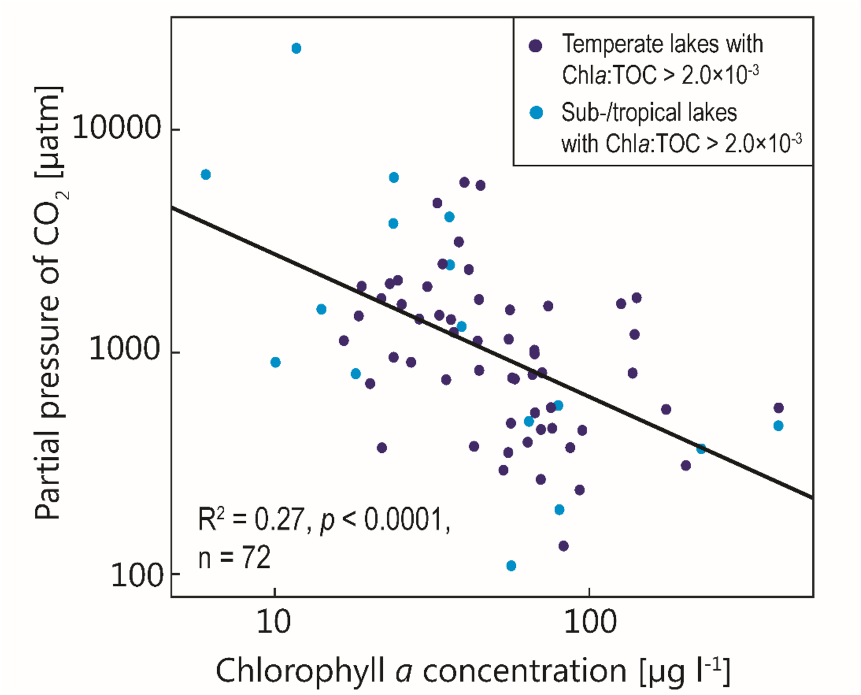

Figure S2. Relationship between the partial pressure of CO_2 (pCO_2) and the chlorophyll a concentration (Chla) in 56 lakes from the temperate, and 16 lakes from the sub-/tropical region. Each data point represents one lake. Both axes are displayed on a logarithmic scale. For the linear regression analysis, the values for pCO_2 and Chla were log-transformed, since they did not follow a normal distribution. Data sources are provided in Table S3.

The available data for testing the relationship between Chl*a* and pCO_2 in rivers with Chl*a*:TOC ratios > 2.0×10^{-3} was limited. However, a recent study from the Han River basin, Korea (Yoon et al. 2017), in which the average Chl*a*:TOC ratio clearly exceeded 2.0×10^{-3} (we calculated the ratio from Chl*a* and DOC), showed a strong negative relation between Chl*a* and pCO_2 in the river basin (Yoon et al. 2017). In contrast, in a river basin with Chl*a*:TOC ratios < 2.0×10^{-3} (we calculated the ratio from Chl*a* and DOC), Seine basin, France (Marescaux et al. 2018), Chl*a* was slightly positively related to pCO_2, supporting our assumption that the Chl*a*:TOC ratio can even be used as simple proxy to identify rivers with a significant pCO_2 reduction by phytoplankton.

Supplementary Figure

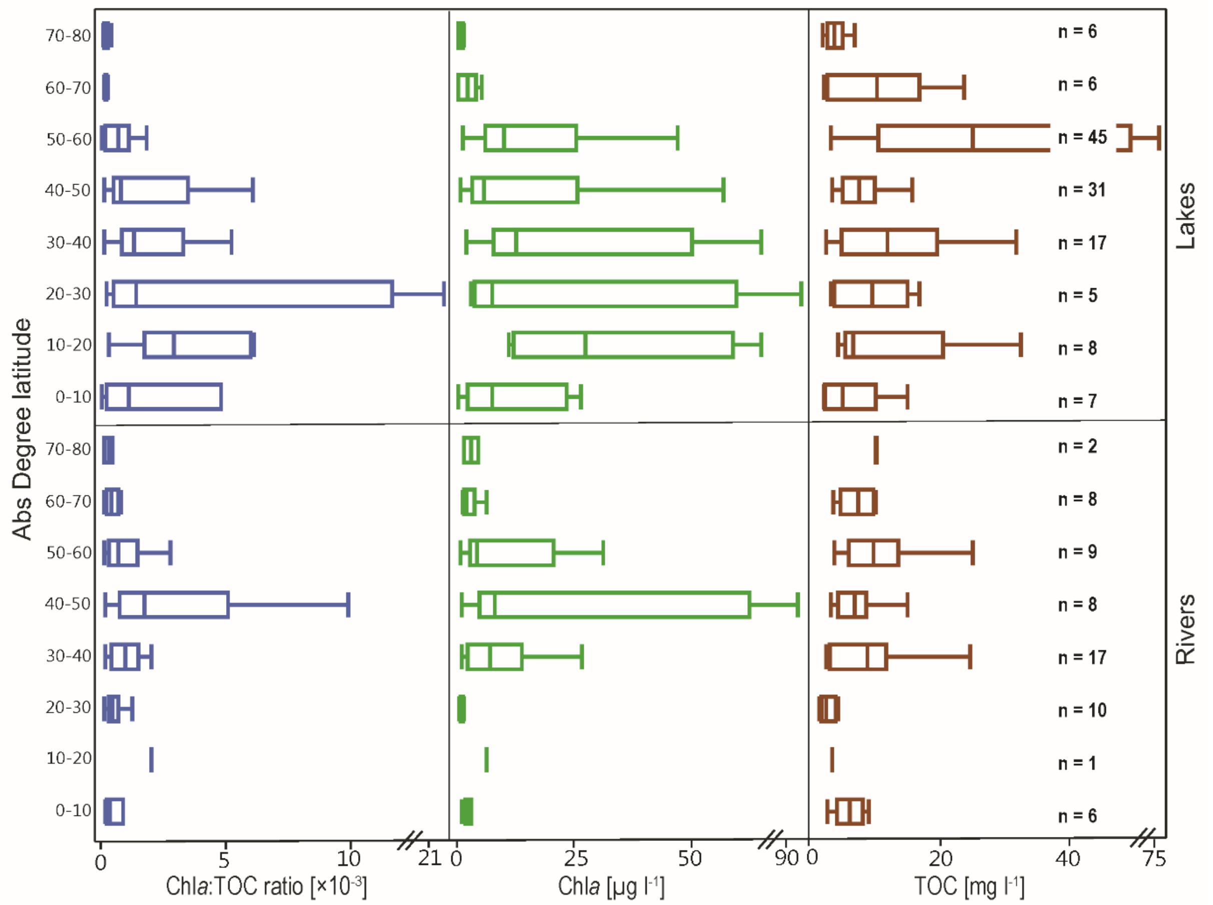

Figure S3. Latitudinal distribution of the mass ratio of chlorophyll a concentration to total organic carbon (Chla:TOC ratio), chlorophyll a concentration (Chla), and total organic carbon concentration (TOC) summarized in latitudinal bands of ten degrees latitude. Shown is the data listed in Tables S1 and S2. The central line represents the median, the ends of the boxes represent the first and the third quartile, and the whiskers extend from the ends of the boxes to the outermost data point that falls within 1.5-times the interquartile range. The sample size (n) is given for each latitudinal band. Data points lying outside of the whiskers (outliers) are not displayed to increase the readability.

Supplementary Tables

Table S1. Water body name, sampling years and months, region of location (region), chlorophyll a concentration (Chla), total organic carbon concentration (TOC), mass ratio of chlorophyll a to total organic carbon (Chla:TOC ratio), and method to derive Chla (Chla method) where "FLU" stands for fluorometric determination, "HPLC" for determination with high-performance liquid chromatography, and "SPM" for spectrophotometric determination, for 61 river sites collected from published literature and two data bases. The Swedish national lake inventory program is abbreviated as "SNLIP", and the monitoring program of the German federal state of Rhineland-Palatinate as "MPRP".

Water body name	Sampling years	Sampling months	Region	Chlorophyll *a* [μg l^{-1}]	TOC [mg l^{-1}]	Chl*a*:TOC ratio [$\times10^{-3}$]	Chl*a* method	Reference
Torne älv	2015, 2016	3 - 10	cold	2.3	9.7	0.2	SPM	SNLIP
Vindelälven	2015, 2016	3 - 10	cold	1.4	3.4	0.4	SPM	SNLIP
Indalsälven	2016	4 - 10	cold	1.6	4.3	0.4	SPM	SNLIP
Ätran River	2015, 2016	3 - 10	temperate	3.5	9.7	0.4	SPM	SNLIP
Göta älv	2015, 2016	3 - 11	temperate	4.3	5	0.9	SPM	SNLIP
Dalälven	2015, 2016	3 - 10	cold	6.3	8	0.8	SPM	SNLIP
Yangtze River	2013, 2014	3, 5, 8, 11	sub/-tropical	1.4	1.6	0.9	SPM	Liu et al. 2016
Yangtze River	2013, 2014	3, 5, 8, 11	sub/-tropical	1.1	2	0.6	SPM	Liu et al. 2016
Yangtze River	2013, 2014	3, 5, 8, 11	sub/-tropical	0.9	1.4	0.6	SPM	Liu et al. 2016
Yangtze River	2013, 2014	3, 5, 8, 11	sub/-tropical	1	2.7	0.4	SPM	Liu et al. 2016
Yangtze River	2013, 2014	3, 5, 8, 11	sub/-tropical	1	2.4	0.4	SPM	Liu et al. 2016
Yangtze River	2013, 2014	3, 5, 8, 11	sub/-tropical	1.5	3.6	0.4	SPM	Liu et al. 2016
Yangtze River	2013, 2014	3, 5, 8, 11	sub/-tropical	1.2	3.1	0.4	SPM	Liu et al. 2016
Daduhe River	2013, 2014	3, 5, 8, 11	sub/-tropical	0.7	2.1	0.3	SPM	Liu et al. 2016
Min Jiang River	2013, 2014	3, 5, 8, 11	sub/-tropical	0.5	4.1	0.1	SPM	Liu et al. 2016
Wu Jiang River	2013, 2014	3, 5, 8, 11	sub/-tropical	0.7	1.3	0.5	SPM	Liu et al. 2016
Xiang Jiang River	2013, 2014	3, 5, 8, 11	sub/-tropical	1.1	3.7	0.3	SPM	Liu et al. 2016

Han Jiang River	2013, 2014	3, 5, 8, 11	sub/-tropical	2.6	2.7	1.0	SPM	Liu et al. 2016
Gan Jiang River	2013, 2014	3, 5, 8, 11	sub/-tropical	1.3	3.6	0.4	SPM	Liu et al. 2016
Saar River	2015	4 - 9	sub/-tropical	8	4.4	1.8		MPRP
Congo River	2013, 2014		sub/-tropical	2.4	9	0.3	HPLC	Descy et al. 2017
Amazon River, Óbidos	2010 - 2012		sub/-tropical	2.3	5.4	0.4	SPM	Ward et al. 2015
Tapajós River	2010 - 2013		sub/-tropical	5.2	2.5	2.1	SPM	Ward et al. 2015
Tocantins River	2010 - 2014		sub/-tropical	2.2	4.5	0.5	SPM	Ward et al. 2015
St. Johns River	1999 - 2001		temperate	18.4	14.6	1.3	SPM	Ouyang 2005
St. Johns River	1999 - 2001		temperate	16.4	16.7	1.0	SPM	Ouyang 2005
St. Johns River	1999 - 2001		temperate	3.33	2.78	1.2	SPM	Ouyang 2005
Upper Ohio River	1998 - 2000	8,9,10	temperate	2	10.8	0.2	FLU	Jack et al. 2002
Kentucky River	1998 - 2000	8,9,10	temperate	9	9.1	1.0	FLU	Jack et al. 2002
Middle Ohio River	1998 - 2000	8,9,10	temperate	4	11.4	0.4	FLU	Jack et al. 2002
Green River	1998 - 2000	8,9,10	temperate	7	13.1	0.5	FLU	Jack et al. 2002
Wabash River	1998 - 2000	8,9,10	temperate	50	24.6	2.0	FLU	Jack et al. 2002
Cumberland River	1998 - 2000	8,9,10	temperate	22	12.1	1.8	FLU	Jack et al. 2002
Tennessee River	1998 - 2000	8,9,10	temperate	8	8.7	0.9	FLU	Jack et al. 2002
Lower Ohio River	1998 - 2000	8,9,10	temperate	10	9.7	1.0	FLU	Jack et al. 2002
Lena River	1989 - 1991	yearly average	cold	4.5	10.2	0.4	n/a	Cauwet & Sidorov 1996
San Joaquin River	2000, 2001	6-11	temperate	26.8	5.5	4.9	FLU	Kratzer et al. 2004

Po River	1995, 1996	all months	temperate	7.9	4	2.0	SPM	Pettine et al. 1998
Pu River	2012, 2013	all months	temperate	92	15	6.1	n/a	Gao et al. 2015
Chena River	2005, 2006	one year	cold	4.1	5.5	0.8	SPM	Cai et al. 2008
Loire River	1982 - 1985	all months	temperate	80	8.1	9.9	SPM	Meybeck et al. 1988
Oyapock River	2014-2016	all months	sub/-tropical	1.09	7.6	0.3	FLU	Gallay et al. 2017
Maroni River	2014-2016	all months	sub/-tropical	1.98	8.7	1.2	FLU	Gallay et al. 2017
Danube River	1995-1996	all months	temperate	10	7.7	0.6	HPLC	Tockner et al. 1999
Morlaix River	1979-1980	all months	temperate	4.7	16.3	1.9	FLU	Wafar et al. 1989
River Warnow	2002	4 - 10	temperate	31.3	6.5	0.1	SPM	Freese et al. 2006
Great Whale River	1990, 1991	all months	cold	0.8	2.8	4.1	SPM	Hudon et al. 1996
York River	2000	3, 10	temperate	11.5	3.2	2.0	FLU	McCallister et al. 2006
Gautami Godavari	2001	5, 6	sub/-tropical	6.4	25.1	2.8	FLU	Bouillon et al. 2003
Scheldt River	1996 - 1998	all months	temperate	69.2	3.7	0.7	FLU	Abril et al. 2002
Rhine	1996 -1998	3, 7, 10, 11	temperate	2.5	5.7	0.2	FLU	Abril et al. 2002
Gironde	1996 - 1998	2, 6, 9, 11	temperate	1.1	9.3	0.3	FLU	Abril et al. 2002
Thames	1996, 1999	2, 9	temperate	2.8	6.9	0.5	FLU	Abril et al. 2002
Sado	1996, 1997	4, 9	temperate	3.2	10.9	0.4	FLU	Abril et al. 2002
Elbe	1997	4	temperate	4.5	9.6	1.0	FLU	Abril et al. 2002
Ems	1997	7	temperate	9.8	3.0	1.7	FLU	Abril et al. 2002
Douro	1997	9	temperate	5	10.0	0.2	FLU	Abril et al. 2002
Yenisey	2003	8	cold	1.47	10.0	0.1	n/a	Hessen et al. 2010

Ob	2005	9	cold	1.14	9.3	0.2	n/a	Hessen et al. 2010
Yukon River	2002	5 - 9	cold	1.78	6.6	0.5	n/a	Guéguen et al. 2006
Mackenzie River	2009	8	cold	3.1	7.6	0.3	n/a	Ortega-Retuerta et al. 2013

Table S2. Water body name, sampling years and months, region of location (region), chlorophyll a concentration (Chla), total organic carbon concentration (TOC), mass ratio of chlorophyll a to total organic carbon (Chla:TOC ratio), and method to derive Chla (Chla method) where "FLU" stands for fluorometric determination, "HPLC" for determination with high-performance liquid chromatography, and "SPM" for spectrophotometric determination, for 125 lake and reservoir sites collected from published literature. Sites marked with an asterisk (*) are average values from clusters of more than 25 lakes from a single province.

**Water body name**	**Sampling years**	**Sampling months**	**Region**	**Chlorophyll *a* [μg l^{-1}]**	**TOC [mg l^{-1}]**	**Chl*a*:TOC ratio [×10^{-3}]**	**Chl*a* method**	**Reference**
Jialing Jiang	2013, 2014	3,5,8,11	sub-/tropical	4.7	3.0	1.6	SPM	Liu et al. 2016
Allgjuttern	1997 - 2006	5 - 10	temperate	2.3	7.0	0.3	SPM	Khalili & Weyhenmeyer 2009
Brunnsjön	1997 - 2006	5 - 10	temperate	3.3	18.0	0.2	SPM	Khalili & Weyhenmeyer 2009
Fiolen	1997 - 2006	5 - 10	temperate	5.2	7.0	0.8	SPM	Khalili & Weyhenmeyer 2009
Fräcksjön	1997 - 2006	5 - 10	temperate	6.1	9.0	0.7	SPM	Khalili & Weyhenmeyer 2009
Härsvatten	1997 - 2006	5 - 10	temperate	1.3	3.0	0.4	SPM	Khalili & Weyhenmeyer 2009
Remmarsjön	1997 - 2006	5 - 10	cold	2.4	10.0	0.2	SPM	Khalili & Weyhenmeyer 2009
Rotehogstjärnen	1997 - 2006	5 - 10	temperate	8.1	12.0	0.7	SPM	Khalili & Weyhenmeyer 2009
Stora Envättern	1997 - 2006	5 - 10	temperate	3.3	10.0	0.3	SPM	Khalili & Weyhenmeyer 2009
St Skärsjön	1997 - 2006	5 - 10	temperate	4.0	4.0	1.0	SPM	Khalili & Weyhenmeyer 2009
Black Hawk			temperate	71.0	11.7	6.1	n/a	Pacheco et al. 2014
Lake Darling			temperate	44.0	10.5	4.2	n/a	Pacheco et al. 2014
Green Valley			temperate	41.0	9.3	4.4	n/a	Pacheco et al. 2014
Lizard Lake			temperate	138.0	34.1	4.1	n/a	Pacheco et al. 2014

Prairie Rose			temperate	49.0	8.7	5.6	n/a	Pacheco et al. 2014
Lake Jaroslawieckie	2000, 2001	3,5,6,7,11	temperate	13.2	12.3	1.1	SPM	Pelechaty et al. 2003
Lakes on Banks Island*	2000	6,7	cold	1.6	6.7	0.2	n/a	Lim et al. 2005
Lake Apopka	1994, 1995		temperate	96.0	4.4	21.7	n/a	Gu et al. 2004
Lakes on Victoria Island*	1997	7	cold	0.4	1.7	0.2	n/a	Michelutti et al. 2002
Polesie Lubelskie Lakes	2004- 2006	4 -11	temperate	68.0	5.6	12.1	SPM	Mieczan 2010
Naroch Lakes			temperate	25.0	9.6	2.6	n/a	Ostapenia et al. 2009
Lake Kinneret			sub-/tropical	18.0	5.1	3.5	n/a	Ostapenia et al. 2009
Lake Ladoga			cold	2.3	10.3	0.2	n/a	Ostapenia et al. 2009
Lake Mendota			temperate	50.6	6.3	8.0	n/a	Ostapenia et al. 2009
Lake Mälaren			temperate	18.5	10.8	1.7	n/a	Köhler et al. 2013
Peter and Paul lakes	2014		temperate	6.3	9.2	0.7	n/a	Michelutti et al. 2005
Lostpack Lake			cold	0.3	1.9	0.2	n/a	Michelutti et al. 2005
Moss Lake			cold	0.5	3.6	0.1	n/a	Michelutti et al. 2005
Farkel Lake			cold	0.3	3.8	0.1	n/a	Michelutti et al. 2005
Goose Lake			cold	1.1	2.8	0.4	n/a	Michelutti et al. 2005
Perfection Lake			cold	0.3	2.7	0.1	n/a	Michelutti et al. 2005
Lake Tanganyka	1975	11	sub-/tropical	2.2	10.1	0.2	FLU	Hecky & Kling 1981
Barr Lake	1991	9, 10	temperate	62.2	11.9	5.2	SPM	Arenz Jr et al. 1996
Bear Creek Reservoir	1991	9, 10	temperate	11.7	4.5	2.6	SPM	Arenz Jr et al. 1996
Carter Lake	1991	9, 10	temperate	0.8	3.8	0.2	SPM	Arenz Jr et al. 1996

Chatfield Reservoir	1991	9, 10	temperate	4.1	3.6	1.1	SPM	Arenz Jr et al. 1996
Cherry Creek Reservoir	1991	9, 10	temperate	9.6	7.5	1.3	SPM	Arenz Jr et al. 1996
Horsetooth Reservoir	1991	9, 10	temperate	3.8	4.2	0.9	SPM	Arenz Jr et al. 1996
Main Reservoir	1991	9, 10	temperate	10.1	6.2	1.6	SPM	Arenz Jr et al. 1996
Standley Lake	1991	9, 10	temperate	2.1	2.3	0.9	SPM	Arenz Jr et al. 1996
Lake Batata	2001, 2002	6, 12	sub-/tropical	16.7	15.1	1.1	SPM	Farjalla et al. 2006
Shingobee Lake	2004		temperate	5.3	5.3	1.0	n/a	Stets et al. 2009
Williams Lake	2004		temperate	4.6	8.2	0.6	n/a	Stets et al. 2009
Patos Lagoon	2002 - 2004	all moths	sub-/tropical	7.6	9.4	0.8	n/a	Oliveira et al. 2006
Lake Dom Helvécio	2000, 2001	dry and rainy season	sub-/tropical	12.6	4.1	3.1	SPM	Petrucio & Barbosa 2004
Lake Carioca	2000, 2001	dry and rainy season	sub-/tropical	64.8	5.1	12.7	SPM	Petrucio & Barbosa 2004
Lake Amarela	2000, 2001	dry and rainy season	sub-/tropical	35.8	6.4	5.6	SPM	Petrucio & Barbosa 2004
Lake Águas Claras	2000, 2001	dry and rainy season	sub-/tropical	41.5	6.8	6.1	SPM	Petrucio & Barbosa 2004
Lake Xolotlán	1987 - 1993		sub-/tropical	65.0	23.3	2.8	SPM	Erikson et al. 1998
Lake Imboassica	1992 - 2005		sub-/tropical	23.2	16.8	1.4	FLU	Marotta et al. 2010
Lake Cabiúnas	1992 - 2005		sub-/tropical	3.0	13.1	0.2	FLU	Marotta et al. 2010
Lake Aguapé	2004, 2005	monthly	sub-/tropical	19.1	11.5	1.7	SPM	Marotta et al. 2012
Lake Barra	2004, 2005	monthly	sub-/tropical	12.0	6.1	2.0	SPM	Marotta et al. 2012
Lake Kivu	2009 - 2011	4, 6, 11	sub-/tropical	2.2	2.1	1.0	HPLC	Morana et al. 2014
Lake Edward	2012	5	sub-/tropical	7.5	5.3	1.4	HPLC	Morana et al. 2014
Lake Albert	2012	5	sub-/tropical	23.5	4.9	4.8	HPLC	Morana et al. 2014
Lake Victoria	2012	5	sub-/tropical	26.5	2.0	13.3	HPLC	Morana et al. 2014

Lake Mangueira	2006, 2007	8, 11, 2, 5	sub-/tropical	6.1	20.8	0.3	SPM	Rodrigues et al. 2015
Lake Biandantang	2003, 2004	monthly	sub-/tropical	20.3	9.2	2.2	SPM	Xing et al. 2006
Lake Taihu	2004	3	sub-/tropical	10.2	13.9	0.7	n/a	Zhang et al. 2005
Lake U60	1993	8	cold	5.3	23.8	0.2	HPLC	Pienitz et al. 2000
Lakes in the Canadian Artic Archipelago*	1979 - 1997		cold	0.6	4.3	0.1	SPM	Hamilton et al. 2001
Utikuma Lakes*	1998	8	cold	29.6	36.1	0.8	SPM	Bayley & Prather 2003
Mistehae Lakes*	1998	8	cold	40.4	27.4	1.5	SPM	Bayley & Prather 2003
Gollinsee	2010 - 2011	all months	temperate	23.0	13.7	1.7	n/a	Brothers et al. 2013
Schulzensee	2010 - 2011	all months	temperate	13.0	12.5	1.0	n/a	Brothers et al. 2013
Santa Olalla	2007	summer	temperate	246.9	82.3	3.0	SPM	De Vicente et al. 2010
Dulce	2007	summer	temperate	594.7	71.3	8.4	SPM	De Vicente et al. 2010
Medina	2007	summer	temperate	37.9	31.7	1.2	SPM	De Vicente et al. 2010
Rincón	2007	summer	temperate	12.6	18.1	0.7	SPM	De Vicente et al. 2010
Zoñar	2007	summer	temperate	16.9	13.1	1.3	SPM	De Vicente et al. 2010
Amarga	2007	summer	temperate	2.5	18.2	0.1	SPM	De Vicente et al. 2010
Bergner	2006	summer	temperate	2.9	7.1	0.4	SPM	De Vicente et al. 2010
Bolger	2006	summer	temperate	8.8	15.0	0.6	SPM	De Vicente et al. 2010
Crampton	2006	summer	temperate	2.9	5.6	0.5	SPM	De Vicente et al. 2010
E Long	2006	summer	temperate	1.5	7.7	0.2	SPM	De Vicente et al. 2010
Hummingbird	2006	summer	temperate	5.9	20.2	0.3	SPM	De Vicente et al. 2010

Inkpot	2006	summer	temperate	5.7	9.8	0.6	SPM	De Vicente et al. 2010
Kickapoo	2006	summer	temperate	5.1	7.3	0.7	SPM	De Vicente et al. 2010
Morris	2006	summer	temperate	10.7	15.8	0.7	SPM	De Vicente et al. 2010
Plum	2006	summer	temperate	18.2	6.5	2.8	SPM	De Vicente et al. 2010
Raspberry	2006	summer	temperate	3.9	7.8	0.5	SPM	De Vicente et al. 2010
Roach	2006	summer	temperate	2.5	3.2	0.8	SPM	De Vicente et al. 2010
Tenderfoot	2006	summer	temperate	3.2	7.3	0.4	SPM	De Vicente et al. 2010
W Long	2006	summer	temperate	1	8.3	0.1	SPM	De Vicente et al. 2010
Ward	2006	summer	temperate	25.7	7.4	3.5	SPM	De Vicente et al. 2010
Võrtsjärv	2008 - 2011		temperate	2.2	18.6	0.1	SPM	Piirsoo et al. 2018
Lake Kasumigaura	1992 - 1993		temperate	65	4.5	14.4	n/a	Imai et al. 2001
Allequash	2000	7, 8	temperate	9.6	4.1	2.3	FLU	Hanson et al. 2003
Big Muskellunge	2000	7, 8	temperate	4.5	5.0	0.9	FLU	Hanson et al. 2003
Little Arbor Vitae	2000	7, 8	temperate	56.9	3.6	16.0	FLU	Hanson et al. 2003
Lake Pääjärvi	2004 - 2005	5, 10, 11	cold	3.6	14.5	0.3	n/a	López Bellido et al. 2009
Teniz	2015	4	temperate	378	56	6.8	SPM	Boros et al. 2017
Sukyrkol	2015	4	temperate	169	51	3.3	SPM	Boros et al. 2017
Kaiyndysor	2015	4	temperate	23	48	0.5	SPM	Boros et al. 2017
Asubastysor	2015	4	temperate	41	66	0.6	SPM	Boros et al. 2017
Ukrash	2015	4	temperate	6	48	0.1	SPM	Boros et al. 2017
Zharsor	2015	4	temperate	9	25	0.4	SPM	Boros et al. 2017

Unknown	2015	5	temperate	10	125	0.1	SPM	Boros et al. 2017
Unknown	2015	5	temperate	8	43	0.2	SPM	Boros et al. 2017
Kaindysor	2015	5	temperate	10	74	0.1	SPM	Boros et al. 2017
Unknown	2015	5	temperate	47	79	0.6	SPM	Boros et al. 2017
Unknown	2015	5	temperate	57	63	0.9	SPM	Boros et al. 2017
Shoshkakol	2014	5	temperate	34	38	0.9	SPM	Boros et al. 2017
Big Aqsuat	2015	4	temperate	12	13	0.9	SPM	Boros et al. 2017
Little Aqsuat	2014	5	temperate	26	25	1.0	SPM	Boros et al. 2017
Unknown	2015	4	temperate	6	48	0.1	SPM	Boros et al. 2017
Unknown	2014	5	temperate	10	19	0.5	SPM	Boros et al. 2017
Zharman Koli	2014	5	temperate	11	32	0.3	SPM	Boros et al. 2017
Zharkol	2014	5	temperate	10	25	0.4	SPM	Boros et al. 2017
Saryqopa Koli	2014	6	temperate	10	61	0.2	SPM	Boros et al. 2017
Little Tengiz	2014	6	temperate	5	109	0.1	SPM	Boros et al. 2017
Kalmakty	2014	6	temperate	5	51	0.1	SPM	Boros et al. 2017
Balyksor	2014	6	temperate	27	24	1.1	SPM	Boros et al. 2017
Boshchesor	2014	6	temperate	8	52	0.2	SPM	Boros et al. 2017
Big Saryoba	2014	6	temperate	9	43	0.2	SPM	Boros et al. 2017
Karasor	2014	6	temperate	13	12	1.1	SPM	Boros et al. 2017
Lake Majcz Wielki			temperate	10.6	7.8	1.4	SPM	Bowszys et al. 2014
Lake Mikolajskie			temperate	17.1	9.5	1.8	SPM	Bowszys et al. 2014

Omerli reservoir	2002 - 2004	all moths	temperate	9.4	3.3	2.9	SPM	Morkoc et al. 2009
Lake Piaseczno	2005 -2006	7 ,8, 9	temperate	9.2	5.1	1.8	n/a	Mieczan 2008
Cajas National Park lakes*	2011 - 2012	8, 9, 10	sub-/tropical	0.21	4.2	0.1	HPLC	Van Colen et al. 2017
Cointzio Reservoir	2009	all moths	sub-/tropical	11	32.6	0.3	SPM	Némery et al. 2016

Table S3. Water body name or lake-ID, region of location (region), chlorophyll a concentration (Chla), total organic carbon concentration (TOC), partial pressure of CO_2 (pCO_2), and mass ratio of chlorophyll a to total organic carbon (Chla:TOC ratio), for 16 sub-/tropical lakes, and 56 temperate lakes collected from published literature and from the Swedish national lake inventory program abbreviated as "SNLIP". These lakes were used for the calculations shown in Figure S2.

**Water body name or lake-EU-ID**	**Region**	**Chlorophyll *a* [µg l^{-1}]**	**TOC [mg l^{-1}]**	***p*CO_2 [µatm]**	**Chl*a*:TOC ratio [$\times 10^{-3}$]**	**Reference**
SE662246-164646	temperate	136.8	16.2	805	8.4	SNLIP
SE659479-163100	temperate	73.7	9.8	1611	7.5	SNLIP
SE620062-135224	temperate	75.2	10.5	564	7.2	SNLIP
SE616415-136415	temperate	94.6	13.6	446	7.0	SNLIP
SE617666-135851	temperate	56.1	8.1	480	6.9	SNLIP
SE662767-166446	temperate	82.5	12.3	135	6.7	SNLIP
SE658198-165237	temperate	66.7	12.7	981	5.3	SNLIP
SE619626-135565	temperate	55	10.7	354	5.1	SNLIP
SE664223-165415	temperate	174.8	34.3	554	5.1	SNLIP
SE616267-136857	temperate	75.9	15.0	455	5.1	SNLIP
SE616333-136689	temperate	55.2	10.9	1142	5.1	SNLIP
SE654832-158701	temperate	66.6	13.6	1019	4.9	SNLIP
SE662674-164394	temperate	65.7	13.6	794	4.8	SNLIP
SE654491-160230	temperate	53.1	12.2	295	4.4	SNLIP
SE659706-163325	temperate	63.4	15.0	394	4.2	SNLIP
SE663072-164112	temperate	69.8	16.7	268	4.2	SNLIP
SE615464-134175	temperate	92.8	22.6	241	4.1	SNLIP
SE660010-161773	temperate	41.2	10.1	2352	4.1	SNLIP
SE655920-164557	temperate	39.9	10.0	5801	4.0	SNLIP
SE659771-162546	temperate	43.95	11.5	1121	3.8	SNLIP
SE653703-159331	temperate	42.8	11.5	378	3.7	SNLIP
SE620985-136408	temperate	55.7	16.0	1548	3.5	SNLIP
SE656833-162888	temperate	36.15	10.4	1401	3.5	SNLIP
SE657393-163395	temperate	28.65	8.5	1408	3.4	SNLIP
SE615767-134254	temperate	86.6	27.1	373	3.2	SNLIP
SE615375-137087	temperate	27	8.5	900	3.2	SNLIP
SE616114-134263	temperate	125.7	39.5	1650	3.2	SNLIP
SE656092-160258	temperate	34.9	11.3	753	3.1	SNLIP

SE616141-133891	temperate	56.6	19.5	768	2.9	SNLIP
SE 662114-166021	temperate	44.45	16.2	829	2.7	SNLIP
SE663919-166636	temperate	33.2	12.1	1465	2.7	SNLIP
SE664222-166844	temperate	69.9	25.9	450	2.7	SNLIP
SE654408-159943	temperate	32.7	12.1	4680	2.7	SNLIP
SE615365-134524	temperate	23.75	8.9	948	2.7	SNLIP
SE656432-160826	temperate	30.4	11.6	1970	2.6	SNLIP
SE658548-162439	temperate	18.8	7.2	1977	2.6	SNLIP
SE620184-139120	temperate	18.4	7.3	1454	2.5	SNLIP
SE663894-161926	temperate	24.5	9.9	2102	2.5	SNLIP
SE654362-159722	temperate	44.9	18.9	5621	2.4	SNLIP
SE658444-132483	temperate	21.7	9.3	1741	2.3	SNLIP
SE662994-166164	temperate	38.3	16.5	3132	2.3	SNLIP
SE653690-162187	temperate	25.2	11.4	1640	2.2	SNLIP
SE656949-164064	temperate	21.8	9.9	372	2.2	SNLIP
SE656365-162404	temperate	44.4	20.4	1725	2.2	SNLIP
SE660011-164920	temperate	34	15.7	2491	2.2	SNLIP
SE658281-163532	temperate	23.1	11.2	2030	2.1	SNLIP
SE660523-160785	temperate	16.5	8.0	1126	2.1	SNLIP
SE654145-161816	temperate	20	9.7	723	2.1	SNLIP
SE661966-164781	temperate	36.9	18.3	1227	2.0	SNLIP
Lake Apopka	sub/-tropical	80	31.4	196	2.6	Gu et al. 2011
Pengxi River-reservoir	sub/-tropical	10	3.5	900	2.9	Huang et al. 2017
Lake Peri	sub/-tropical	18	6.4	800	2.8	Fontes et al. 2015
Lake Carioca	sub/-tropical	64	5.1	489	12.6	Reis & Barbosa 2014, Petrucio & Barbosa 2004
Three Gorges Reservoir	sub/-tropical	14	1.4	1559	10.0	Li et al. 2017
Lake Janauacá	sub/-tropical	23.8	5.2	6102	4.6	Amaral et al. 2018
Genipapo Lake	sub/-tropical	35.8	9.4	4057	3.8	Ulloa 2004
Osmar Lake	sub/-tropical	11.7	5.2	23249	2.2	Ulloa 2005
Bilé lake	sub/-tropical	6	2.3	6289	2.6	Ulloa 2006

Peregrina	temperate	202.1	13.3	310	15.2	Kosten et al. 2010
El Paraiso	temperate	398.7	32.8	562	12.2	Kosten et al. 2010
de Oviedo	temperate	138.8	16.3	1201	8.5	Kosten et al. 2010
del Holandés	temperate	140.9	27.2	1756	5.2	Kosten et al. 2010
Piorno	sub/-tropical	397.8	79.6	467	5.0	Kosten et al. 2010
Estancia Bellavista	sub/-tropical	226.1	49.8	368	4.5	Kosten et al. 2010
Laguna Blanca	sub/-tropical	56.2	13.0	110	4.3	Kosten et al. 2010
Santa Barbara	temperate	70.5	17.6	808	4.0	Kosten et al. 2010
Acude das Lajes Pintadas	sub/-tropical	79.3	24.9	576	3.2	Kosten et al. 2010
Laguna del Barron	temperate	57.7	18.8	759	3.1	Kosten et al. 2010
Lagoa sem nome 2	sub/-tropical	23.7	9.1	3789	2.6	Kosten et al. 2010
do Mato	sub/-tropical	35.9	13.9	2466	2.6	Kosten et al. 2010
Acude Recreio	sub/-tropical	39.1	15.9	1304	2.5	Kosten et al. 2010
El Bagual	temperate	66.9	27.9	534	2.4	Kosten et al. 2010

References

Abril G, Nogueira M, Etcheber H, Cabeçadas G, Lemaire E, Brogueira M (2002) Behaviour of organic carbon in nine contrasting European estuaries. Estuarine, Coastal and Shelf science 54:241-262

Amaral JHF et al. (2018) Influence of plankton metabolism and mixing depth on CO2 dynamics in an Amazon floodplain lake. Science of the Total Environment 630:1381-1393

Arenz Jr R, Lewis Jr W, Saunders J (1996) Determination of chlorophyll and dissolved organic carbon from reflectance data for Colorado reservoirs. International Journal of Remote Sensing 17:1547-1565

Bayley SE, Prather CM (2003) Do wetland lakes exhibit alternative stable states? Submersed aquatic vegetation and chlorophyll in western boreal shallow lakes. Limnology and Oceanography 48:2335-2345

Boros E, Jurecska L, Tatár E, Vörös L, Kolpakova M (2017) Chemical composition and trophic state of shallow saline steppe lakes in central Asia (North Kazakhstan). Environmental Monitoring and Assessment 189:546

Bouillon S et al. (2003) Inorganic and organic carbon biogeochemistry in the Gautami Godavari estuary (Andhra Pradesh, India) during pre-monsoon: The local impact of extensive mangrove forests. Global Biogeochemical Cycles 17:1114

Bowszys M, Dunalska J, Jaworska B (2014) Zooplankton response to organic carbon level in lakes of differing trophic states. Knowledge and Management of Aquatic Ecosystems:10

Brothers SM et al. (2013) A regime shift from macrophyte to phytoplankton dominance enhances carbon burial in a shallow, eutrophic lake. Ecosphere 4:1-17

Cai Y, Guo L, Douglas TA (2008) Temporal variations in organic carbon species and fluxes from the Chena River, Alaska. Limnology and Oceanography 53:1408-1419

Cauwet G, Sidorov I (1996) The biogeochemistry of Lena River: organic carbon and nutrients distribution. Marine Chemistry 53:211-227

De Vicente I, Ortega-Retuerta E, Mazuecos IP, Pace ML, Cole JJ, Reche I (2010) Variation in transparent exopolymer particles in relation to biological and chemical factors in two contrasting lake districts. Aquatic Sciences 72:443-453

Engel F, Drakare S, Weyhenmeyer GA (2019) Environmental conditions for phytoplankton influenced carbon dynamics in boreal lakes. Aquatic Sciences 81:35 doi:10.1007/s00027-019-0631-6

Erikson R, Hooker E, Mejia M, Zelaya A, Vammen K (1998) Optimal conditions for primary production in a polymictic tropical lake (Lake Xolotlán, Nicaragua). Hydrobiologia 382:1-16

Farjalla VF, Azevedo DA, Esteves FA, Bozelli RL, Roland F, Enrich-Prast A (2006) Influence of hydrological pulse on bacterial growth and DOC uptake in a clear-water Amazonian lake. Microbial Ecology 52:334-344

Fontes ML, Marotta H, MacIntyre S, Petrucio M (2015) Inter-and intra-annual variations of p CO2 and p O2 in a freshwater subtropical coastal lake. Inland Waters 5:107-116

Freese H, Karsten U, Schumann R (2006) Bacterial abundance, activity, and viability in the eutrophic river Warnow, Northeast Germany. Microbial Ecology 51:117-127

Gallay M et al. (2018) Dynamics and fluxes of organic carbon and nitrogen in two Guiana Shield river basins impacted by deforestation and mining activities. Hydrological Processes 32:17-29

Gao H et al. (2015) Chemometrics data of water quality and environmental heterogeneity analysis in Pu River, China. Environmental Earth Sciences 73:5119-5129

Gu B, Schelske CL, Coveney MF (2011) Low carbon dioxide partial pressure in a productive subtropical lake. Aquatic Sciences 73:317-330

Gu B, Schelske CL, Hodell DA (2004) Extreme13 C enrichments in a shallow hypereutrophic lake: Implications for carbon cycling. Limnology and Oceanography 49:1152-1159

Guéguen C, Guo L, Wang D, Tanaka N, Hung C-C (2006) Chemical characteristics and origin of dissolved organic matter in the Yukon River. Biogeochemistry 77:139-155

Hamilton PB, Gajewski K, Atkinson DE, Lean DR (2001) Physical and chemical limnology of 204 lakes from the Canadian Arctic Archipelago. Hydrobiologia 457:133-148

Hanson PC, Bade DL, Carpenter SR, Kratz TK (2003) Lake metabolism: relationships with dissolved organic carbon and phosphorus. Limnology and Oceanography 48:1112-1119

Hecky R, Kling H (1981) The phytoplankton and protozooplankton of the euphotic zone of Lake Tanganyika: Species composition, biomass, chlorophyll content, and spatio-temporal distribution 1. Limnology and Oceanography 26:548-564

Hessen DO, Carroll J, Kjeldstad B, Korosov AA, Pettersson LH, Pozdnyakov D, Sørensen K (2010) Input of organic carbon as determinant of nutrient fluxes, light climate and productivity in the Ob and Yenisey estuaries. Estuarine, Coastal and Shelf Science 88:53-62

Huang Y, Yasarer LM, Li Z, Sturm BS, Zhang Z, Guo J, Shen Y (2017) Air–water CO 2 and CH 4 fluxes along a river–reservoir continuum: Case study in the Pengxi River, a tributary of the Yangtze River in the Three Gorges Reservoir, China. Environmental monitoring and assessment 189:223

Hudon C, Morin R, Bunch J, Harland R (1996) Carbon and nutrient output from the Great Whale River (Hudson Bay) and a comparison with other rivers around Quebec. Canadian Journal of Fisheries and Aquatic Sciences 53:1513-1525

Imai A, Fukushima T, Matsushige K, Kim YH (2001) Fractionation and characterization of dissolved organic matter in a shallow eutrophic lake, its inflowing rivers, and other organic matter sources. Water Research 35:4019-4028

Jack J, Sellers T, Bukaveckas PA (2002) Algal production and trihalomethane formation potential: an experimental assessment and inter-river comparison. Canadian Journal of Fisheries and Aquatic Sciences 59:1482-1491

Khalili MI, Weyhenmeyer GA (2009) Growing season variability of nitrate along a trophic gradient–contrasting patterns between lakes and streams. Aquatic Sciences 71:25-33

Köhler SJ, Kothawala D, Futter MN, Liungman O, Tranvik L (2013) In-lake processes offset increased terrestrial inputs of dissolved organic carbon and color to lakes. PloS one 8:e70598

Kosten S et al. (2010) Climate-dependent CO2 emissions from lakes. Global Biogeochemical Cycles 24:GB2007 doi:10.1029/2009GB003618

Kratzer CR, Dileanis PD, Zamora C, Silva SR, Kendall C, Bergamaschi BA, Dahlgren RA (2004) Sources and transport of nutrients, organic carbon, and chlorophyll-a in the San Joaquin River upstream of Vernalis, California, during summer and fall, 2000 and 2001. Water-Resources Investigations Report 3:4127

Li S, Wang F, Luo W, Wang Y, Deng B (2017) Carbon dioxide emissions from the Three Gorges Reservoir, China. Acta Geochimica 36:645-657

Lim DS, Douglas MS, Smol JP (2005) Limnology of 46 lakes and ponds on Banks Island, NWT, Canadian Arctic archipelago. Hydrobiologia 545:11-32

Liu S, Lu XX, Xia X, Zhang S, Ran L, Yang X, Liu T (2016) Dynamic biogeochemical controls on river pCO2 and recent changes under aggravating river impoundment: An example of the subtropical Yangtze River. Global Biogeochemical Cycles 30:880-897

López Bellido J, Tulonen T, Kankaala P, Ojala A (2009) CO2 and CH4 fluxes during spring and autumn mixing periods in a boreal lake (Pääjärvi, southern Finland). Journal of Geophysical Research: Biogeosciences 114

Marescaux A, Thieu V, Borges AV, Garnier J (2018) Seasonal and spatial variability of the partial pressure of carbon dioxide in the human-impacted Seine River in France. Scientific Reports 8:13961

Marotta H, Duarte CM, Meirelles-Pereira F, Bento L, Esteves FA, Enrich-Prast A (2010) Long-term CO 2 variability in two shallow tropical lakes experiencing episodic eutrophication and acidification events. Ecosystems 13:382-392

Marotta H, Fontes M, Petrucio M (2012) Natural events of anoxia and low respiration index in oligotrophic lakes of the Atlantic Tropical Forest. Biogeosciences 9:2879–2887

McCallister SL, Bauer JE, Ducklow HW, Canuel EA (2006) Sources of estuarine dissolved and particulate organic matter: a multi-tracer approach. Organic Geochemistry 37:454-468

Meybeck M, Cauwet G, Dessery S, Somville M, Gouleau D, Billen G (1988) Nutrients (organic C, P, N, Si) in the eutrophic river Loire (France) and its estuary. Estuarine, Coastal and Shelf Science 27:595-624

Michelutti N, Douglas MS, Lean DR, Smol JP (2002) Physical and chemical limnology of 34 ultra-oligotrophic lakes and ponds near Wynniatt Bay, Victoria Island, Arctic Canada. Hydrobiologia 482:1-13

Michelutti N, Wolfe AP, Vinebrooke RD, Rivard B, Briner JP (2005) Recent primary production increases in arctic lakes. Geophysical Research Letters 32

Mieczan T (2008) Diversity and vertical distribution of planktonic ciliates in a stratified mesotrophic lake: relationship to environmental conditions. Oceanological and Hydrobiological Studies 37:83-95

Mieczan T (2010) Periphytic ciliates in three shallow lakes in eastern Poland: a comparative study between a phytoplankton-dominated lake, a phytoplankton-macrophyte lake and a macrophyte-dominated lake. Zoological Studies 49:589-600

Morana C, Sarmento H, Descy J-P, Gasol JM, Borges AV, Bouillon S, Darchambeau F (2014) Production of dissolved organic matter by phytoplankton and its uptake by heterotrophic prokaryotes in large tropical lakes. Limnology and Oceanography 59:1364-1375

Morkoç E, Tüfekçi V, Tüfekçi H, Tolun L, Karakoç FT, Güvensel T (2009) Effects of land-based sources on water quality in the Omerli reservoir (Istanbul, Turkey). Environmental Geology 57:1035-1045

Némery J, Gratiot N, Doan P, Duvert C, Alvarado-Villanueva R, Duwig C (2016) Carbon, nitrogen, phosphorus, and sediment sources and retention in a small eutrophic tropical reservoir. Aquatic Sciences 78:171-189

Oliveira JL, Boroski M, Azevedo JC, Nozaki J (2006) Spectroscopic investigation of humic substances in a tropical lake during a complete hydrological cycle. Acta Hydrochimica et Hydrobiologica 34:608-617

Ortega-Retuerta E, Joux F, Jeffrey WH, Ghiglione J-F (2013) Spatial variability of particle-attached and free-living bacterial diversity in surface waters from the Mackenzie River to the Beaufort Sea (Canadian Arctic). Biogeosciences 10:2747-2759

Ostapenia AP, Parparov A, Berman T (2009) Lability of organic carbon in lakes of different trophic status. Freshwater Biology 54:1312-1323

Ouyang Y (2005) Evaluation of river water quality monitoring stations by principal component analysis. Water Research 39:2621-2635

Pacheco FS, Roland F, Downing JA (2014) Eutrophication reverses whole-lake carbon budgets. Inland Waters 4:41-48 doi:10.5268/IW-4.1.614

Pełechaty M, Pełechata A, Niedzielski P (2003) Spatial and temporal variability of TOC concentrations in a shallow and eutrophicated lake ecosystem (Lake Jarosławieckie, Wielkopolski National Park, Western Poland). Polish Journal of Environmental Studies 12:607-611

Petrucio M, Barbosa F (2004) Diel variations of phytoplankton and bacterioplankton production rates in four tropical lakes in the middle Rio Doce basin (southeastern Brazil). Hydrobiologia 513:71-76

Pettine M, Patrolecco L, Camusso M, Crescenzio S (1998) Transport of carbon and nitrogen to the northern Adriatic Sea by the Po River. Estuarine, Coastal and Shelf Science 46:127-142

Pienitz R, Smol JP, Last WM, Leavitt PR, Cumming BF (2000) Multi-proxy Holocene palaeoclimatic record from a saline lake in the Canadian Subarctic. The Holocene 10:673-686

Piirsoo K et al. (2018) Changes in particulate organic matter passing through a large shallow lowland lake. Proceedings of the Estonian Academy of Sciences 67

Reis P, Barbosa F (2014) Diurnal sampling reveals significant variation in CO2 emission from a tropical productive lake. Brazilian Journal of Biology 74:S113-S119

Rodrigues LR, Motta-Marques D, Fontoura NF (2015) Fish community in a large coastal subtropical lake: how an environmental gradient may affect the structure of trophic guilds. Limnetica 34:495-506

Stets EG, Striegl RG, Aiken GR, Rosenberry DO, Winter TC (2009) Hydrologic support of carbon dioxide flux revealed by whole-lake carbon budgets. Journal of Geophysical Research-Biogeosciences 114 doi:10.1029/2008jg000783

Tockner K, Pennetzdorfer D, Reiner N, Schiemer F, Ward J (1999) Hydrological connectivity, and the exchange of organic matter and nutrients in a dynamic river–floodplain system (Danube, Austria). Freshwater Biology 41:521-535

Ulloa V (2004) Density and biomass of planktonic rotifers in different habitats in upper Parana River(PR, Brazil). Acta Limnologica Brasiliensia 16:281-292

Van Colen WR et al. (2017) Limnology and trophic status of glacial lakes in the tropical Andes (Cajas National Park, Ecuador). Freshwater Biology 62:458-473

Wafar M, Le Corre P, Birrien J (1989) Transport of carbon, nitrogen and phosphorus in a Brittany river, France. Estuarine, Coastal and Shelf Science 29:489-500

Ward ND et al. (2015) The compositional evolution of dissolved and particulate organic matter along the lower Amazon River—Óbidos to the ocean. Marine Chemistry 177:244-256

Xing Y, Xie P, Yang H, Wu A, Ni L (2006) The change of gaseous carbon fluxes following the switch of dominant producers from macrophytes to algae in a shallow subtropical lake of China. Atmospheric Environment 40:8034-8043

Yoon TK, Jin H, Begum MS, Kang N, Park J-H (2017) CO2 outgassing from an urbanized river system fueled by wastewater treatment plant effluents. Environmental Science & Technology 51:10459-10467 doi:10.1021/acs.est.7b02344

Zhang YL, Qin BQ, Chen WM, Zhu GW (2005) A preliminary study of chromophoric dissolved organic matter (CDOM) in Lake Taihu, a shallow subtropical lake in China. Acta Hydrochimica et Hydrobiologica 33:315-323
